# Chronic postoperative inguinal pain (CPIP) after pediatric inguinal hernia repair—a retrospective analysis

**DOI:** 10.1007/s10029-024-03245-z

**Published:** 2025-01-06

**Authors:** A. Widder, H. Bucher, A. K. Reinhold, L. Maroske, T. Meyer, A. Wiegering, J. F. Lock, C. -T. Germer, H. L. Rittner, N. Schlegel, Michael Meir

**Affiliations:** 1 Department of General, Visceral, Transplant, Vascular and Pediatric Surgery, University Hospital of Wuerzburg, Wuerzburg, Germany; 2 Center for Interdisciplinary Pain Medicine, Department of Anesthesiology, Intensive Care Medicine, Emergency Medicine and Pain Therapy, University Hospital of Wuerzburg, Wuerzburg, Germany

**Keywords:** Pediatric hernia repair, Chronic postoperative inguinal pain, Resolved inguinal pain, Inguinal hernia, Cauda epidural block

## Abstract

**Background:**

Surgical treatment of inguinal hernias in children is one of the most common operative procedures worldwide. During surgery for inguinal hernias in adults, chronic pain develops in approximately 10% of all cases. In children, there has been limited research to determine whether they may also develop this chronic postsurgical inguinal pain (CPIP). The aim of this study was to investigate the prevalence of CPIP in children after open inguinal hernia surgery and to identify possible risk factors and protective factors for the development of CPIP.

**Methods:**

A single center retrospective analysis of patients aged 4 to 15 years who underwent inguinal hernia repair from 2020 to 2022 was performed. A detailed analysis based on the local database was used to analyze existing pre-existing conditions, perioperative information and the use of a cauda epidural block. A standardized follow-up questionnaire was used to evaluate the prevalence of CPIP and the duration of postoperative analgesic medication.

**Results:**

A total of 176 cases were included in the detailed analysis. 3.4 % of the children complained CPIP 3 months after surgery with a mean follow-up period of 26.4 months. At the time of the survey, 50% of CPIP patients reported a resolving from chronic pain. Our analyzes showed a potential higher CPIP rate in females (83.3%; p=0.040), older children (8.3 years vs. 5 years; p=0.006) and chronic pain history (16.7% vs. 2.4%; p=0.038).Furthermore, Children mitght profit from a intraoperative cauda epidural block since we observed a lower rate of CPIP (66.7% (4/6) vs. 97% (164/170); p=0.019) in these patients.

**Conclusion:**

We were able to identify initial risk factors such as female gender, older patient age and a history of chronic pain. In addition, we were able to obtain information on possible protective factors such as an intraoperative cauda epidural block and adequate postoperative analgesia. However, further studies are required to clarify the pathogenesis and to confirm predictors and protective factors in order to improve therapeutic approaches.

**Supplementary information:**

The online version contains supplementary material available at 10.1007/s10029-024-03245-z.

## Introduction

Surgical treatment of inguinal hernias in children is one of the most common operative procedures worldwide [1]. In most cases, the hernia is lateral to the epigastric vessels. The incidence of inguinal hernia is higher in preterm infants (9–11%) than in term infants (1–5%) [2, 3]. The surgical treatment of inguinal hernia in children is performed differently to inguinal hernia in adults. Reconstructive procedures with narrowing of the inguinal canal are avoided in order to prevent testicular atrophy. Surgical treatment with using a mesh implant is not common, only in exceptional cases such as recurrence hernias or femoral hernias [4–6]. Moreover, the inguinal hernia repair in children is by default performed as an open surgery. Laparoscopic inguinal hernia repair in children is associated with higher recurrence rates and longer operating times. Laparoscopy only offers advantages in cases of bilateral inguinal hernias, whereby the recurrence rate can be reduced to the rate for open treatment by an experienced surgeon [7].

After inguinal hernia repair in adults, chronic pain develops in approximately 10–14% of cases [8]. Chronic postoperative inguinal pain (CPIP) is defined as pain lasting at least 3 months postoperatively with restrictions in everyday life. Although CPIP resolves within one year postoperatively in up to 70% of cases, 30% of patients suffer from it permanently [9]. In children, research on CPIP after inguinal hernia repair is limited. Retrospective studies found a prevalence of CPIP in children of 2–3%, thus considerably lower than in adults [10, 11]. However, in these studies, patients were followed-up after 16 to 50 years, a period after which pain may have been resolved. Accordingly, one study with a maximum follow-up period of 48 months reported a higher CPIP rate (5.1%) [12]. This reinforces the assumption that CPIP can also resolve over a longer period of time. The purpose of this retrospective study is to investigate the prevalence of CPIP in children after open inguinal hernia repair and possible risk factors for the development of a CPIP.

## Materials and methods

### Study design

All patients aged 4 to 15 years at the time of the survey who underwent inguinal hernia surgery at the Department of Surgery at the University Hospital Würzburg between January 1st 2020 and December 31th 2022 were included in this retrospective single-center data analysis. The minimum age of 4 years at the time of the survey ensured that the children were able to recognize simple pictograms and answer questions, in addition to the anamnesis by the parents [13]. The primary endpoint of the analysis is the incidence of CPIP after inguinal hernia surgery. Secondary endpoints are possible risk factors, the length of hospital stay, the period and intensity of persistent pain postoperatively as well as the duration of analgesic medication postoperatively. The study was approved by the local ethics committee (No. 103/23). Sample size calculations revealed that 96 included patients are necessary to detect a potential prevalence of 2–5% CPIP patients after pediatric inguinal hernia repair (with an α-error of 0.05 and β-error of 0.20).

### Data acquisition

In all cases, clinical data and perioperative information was collected using the local database.

- *Baseline patient characteristics*: gender, age at operation, premature birth, ASA classification of the physical status, Body Mass Index (BMI), pre-existing conditions, pre-existing chronic pain, previous inguinal hernia repair.
- *Perioperative and clinical data*: localization of the hernia, surgical technique (open or laparoscopic), operating time, caudal epidural block, pain medication during hospital stay, length of hospital stay, readmission and analgesics score to enable comparison of postsurgical pain [14].

A postoperative follow-up was performed using a defined questionnaire. This questionnaire contained questions on current pain, pain intensity and duration, as well as information on the length for pain medication postoperatively and days missed at work by the parents due to the child’s illness (Supplemental material 1). All data was collected using the REDCap platform [15]. All Patients with reported CPIP underwent a clinical follow-up to exclude other pathologies such as hernia recurrence.

### Definition of CPIP in this study

In our data analysis, we defined CPIP according to the current EuraHS guidelines as inguinal pain of ≥ 3 on a visual analog scale (VAS) of 0–10 with 0 representing no pain and 10 representing maximal pain lasting more than 3 months postoperatively [16]. In addition, children with CPIP were divided into two subgroups: children with persistent CPIP (pCPIP) at the time of the survey and children with resolved CPIP (rCPIP), who had pain for more than 3 months postoperatively, but no longer any pain at the time of the survey.

### Statistical analysis

Statistical analysis was performed using IBM SPSS 28.0 (IBM SPSS, Armonk, New York, USA). Differences between groups were calculated using Welch’s t-test and Chi^2^-test as well as single factor variance and analysis of covariance or as repeated measure ANOVA. In the case of multiple t-tests, Bonferroni correction was performed account for multiple testing. Significance was set at *p* < 0.05. Descriptive analyses included mean (MV), minimum (min), and maximum (max) values given as range, percentage, and standard deviation.

## Results

### Definition of the study population

**Fig. 1:**
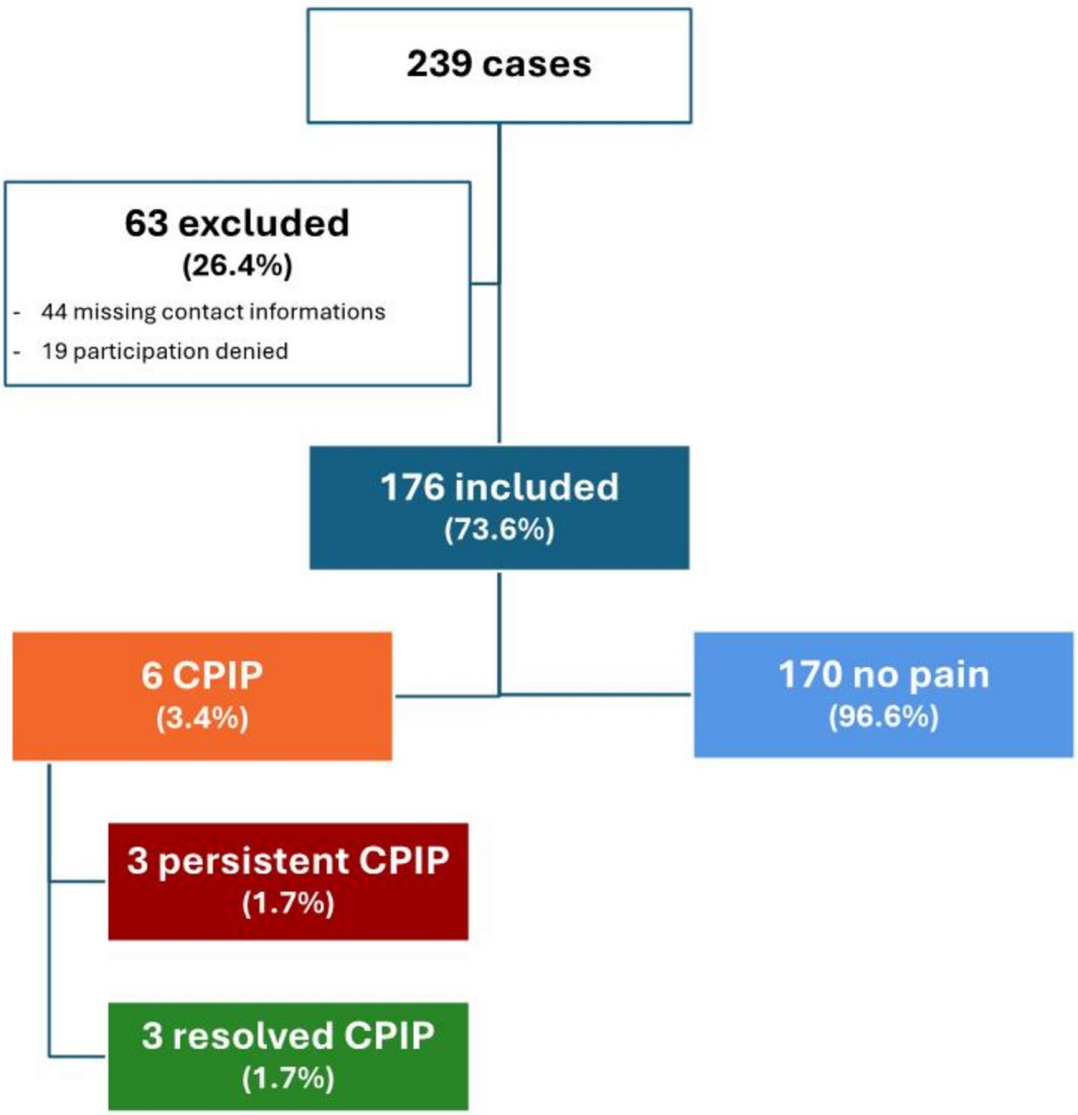
Study population. 176 children were included in the study. Six children developed CPIP following inguinal hernia repair, that resolved in 50% of the patients

239 cases were identified in the years from 2020 to 2022. If both inguinal hernias were operated at two different time points, these patients were regarded as two cases. 63 (26.4%) patients were excluded from the data analysis: 44 patient cases could not be contacted due to changes in contact and address data and in 19 cases participation in the study was refused. The systematic survey was conducted with 176 cases, which corresponds to a response rate of 73.6%. These cases were divided into two groups: Patients with and without CPIP. This showed a CPIP rate of 3.4%. Patients with CPIP were additionally divided into two subgroups. 3 patients (1.7%) showed persistent CPIP (pCPIP) at the time of the survey, 3 patients (1.7%) had pain for more than 3 months postoperatively, but were pain-free again at the time of the survey (resolved CPIP, rCPIP) (Fig. 1). The mean follow-up was 26.4 months with a range of 9 to 44 months. The clinical follow-up did not reveal any surgical problems or hernia recurrences in patients with pCPIP. Therefore, without clinical evidence of other pathologies or hernia recurrences these patients can be assumed to have manifest CPIP.

### Potential risk factors for developing a CPIP

We observed differences between the “CPIP” and “no CPIP” groups in terms of gender, age at surgery, history of chronic pain and surgery with caudal epidural block.

There were significantly more female patients in the CPIP group (4 of 6 patient; *p* = 0.040). Furthermore, the children were older at time of surgery (8.3 years vs. 5 years; *p* = 0.006). The rate of chronic pain history was also significantly higher in the CPIP group. 16.7% of the CPIP patient reported chronic pain in other regions (e.g. back pain) while only 2.4% of the non-CPIP patients suffered from other chronic pain (*p* = 0.038). ASA classification, BMI, premature birth, preexisting conditions and previous inguinal hernia surgery did not differ significantly (Table 1).

**Table 1:** Baseline patient´s characteristics

	NoCPIP = 170 (96.6%)	CPIP = 6 (3.4%)	p-value
Sex			**0.040**
Male n (%)	100 (58.8)	1 (16.7)	
Female n (%)	70 (41.2)	5 (83.3)	
Age at operation [years] mean value (mv) (range)	5.0 (2.8)	8.3 (5.3)	**0.006**
Premature birth n (%)	12 (7.1)	1 (16.7)	0.377
ASA mv (SD)	1.19 (0.4)	1.17 (0.4)	0.899
BMI [kg/m^2^] mv (SD)	15.8 (3.2)	17.9 (3.3)	0.119
Pre-existing conditions n (%)			0.870
Cardiopulmonary	15 (8.8)	0	
Neurological/psychological	7 (4.1)	1 (16.7)	
Urogenital	3 (1.8)	0	
Dermatological/allergological	4 (2.4)	0	
Ophthalmologic/otorhinolaryngologic	2 (1.2)	0	
Developmental disorder	2 (1.2)	0	
Malignancy	2 (1.2)	0	
Syndromal-associated	1 (0.6)	0	
Hemophilia	1 (0.6)	0	
Pre-existing chronic pain n (%)	4 (2.4)	1 (16.7)	**0.038**
Previous inguinal hernia surgery n (%)			0.783
No	155 (91.2)	5 (83.3)	
Yes—ipsilateral side	0	0	
Yes—contralateral side	14 (8.2)	1 (16.7)	
Yes—both sides	1 (0.6)	0	

Caudal epidural block during surgical treatment was less common among patients who later developed CPIP(66.7% vs. 97%; *p* = 0.019). Although not significant, there was a tendency towards a longer operation time in patients with CPIP (43.2 min vs. 26.9 min, *p* = 0.062). The length of hospital stay was significantly longer for patients with CPIP (1.0 days vs. 0.4 days, *p* = 0.018) (Table 2).

**Table 2:** Perioperative and clinical data

	noCPIP = 170 (96.6%)	CPIP = 6 (3.4%)	p-value
Localization of the hernia n (%)			0.466
Left	54 (31.8)	3 (50)	
Right	103 (60.6)	3 (50)	
Both sides	13 (7.6)	0	
Surgical procedure n (%)			0.966
Open	169 (99.4)	6 (100)	
Laparoscopic	1 (0.6)	0	
Operating time [11] MV (SD)	26.9 (20.5)	43.2 (30.3)	0.062
Cauda epidural block n (%)	164 (97)	4 (66.7)	**0.019**
Pain medication n (%)			0.685
No	72 (42.4)	1 (16.7)	
Ibuprofen	57 (33.5)	3 (50)	
Paracetamol	10 (5.9)	0	
Metamizole	2 (1.2)	0	
Opioids	3 (1.8)	0	
Combination	26 (15.3)	2 (33.3)	
Days in hospital MV (SD)	0.4 (0.6)	1.0 (2.0)	**0.018**
Readmission n (%)	1 (0.6)	0	0.966
Analgesics score MV (SD)	0.32 (0.6)	0.50 (0.8)	0.457

### Analgesics score with no impact on developing CPIP

Using the known analgesics score there was no significant differences between the two groups. Patients who developed a CPIP showed a similar analgesic requirement on the day of discharge to patients without CPIP (0.5 vs. 032; *p* = 0.457) (Table 2). However, it should be noted that the operation is usually performed on an outpatient basis.

### Prolonged analgesia as protection against persistent CPIP

**Fig. 2:**
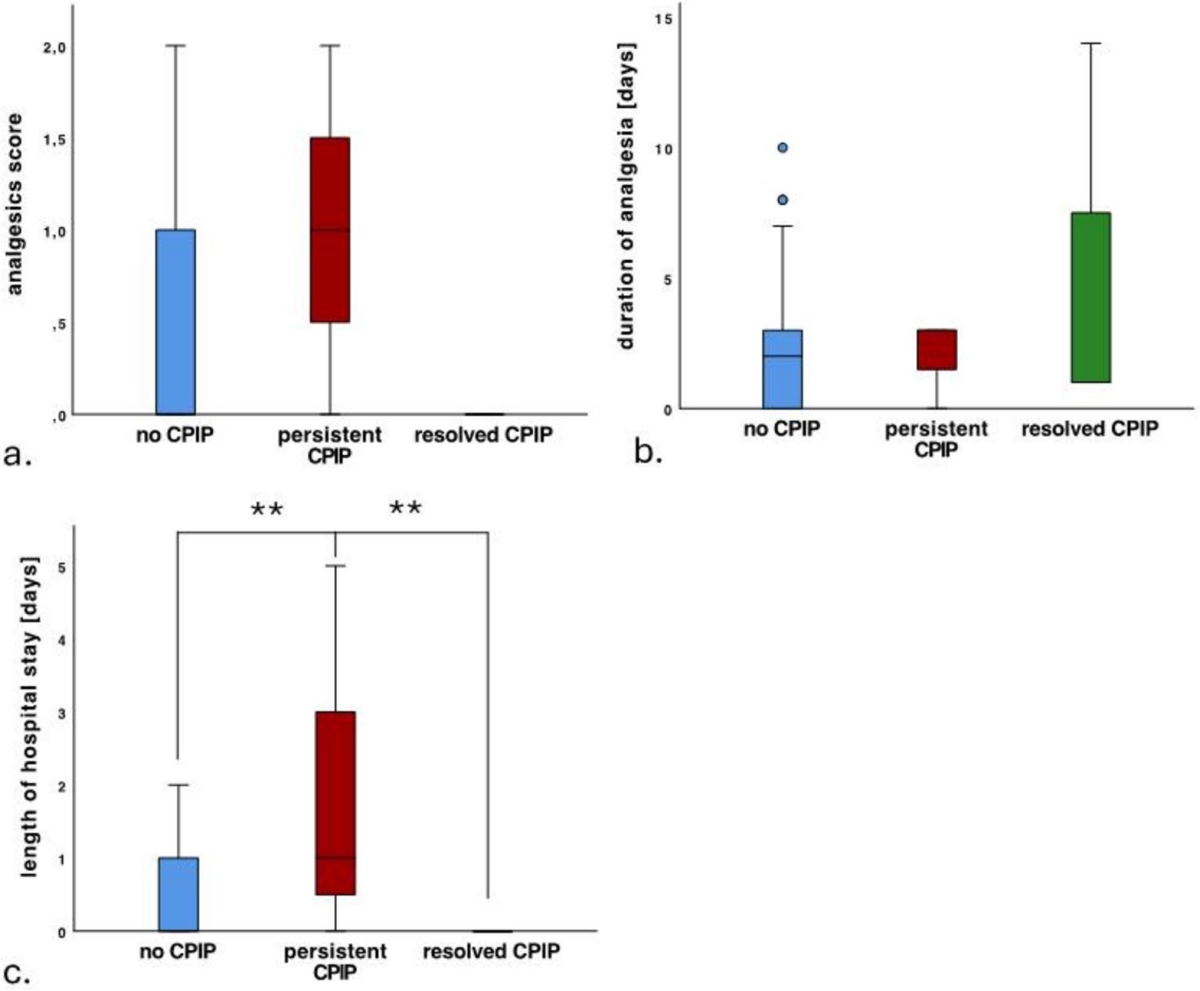
Graphs of analgesics score, duration of analgetic medication and length of stay are shown. While the duration and type of analgetic medication did not differ between the groups, children with CPIP had to stay sig. longer (p< 0.05)

In the subgroup analysis noCPIP versus pCPIP versus rCPIP, the analgesics score showed no significant differences. However, patients with pCPIP tended to have a higher score (1.0 vs. rCPIP 0.0 vs. noCPIP 0.32; *p* = 0.086) (Fig. 2a). Furthermore, the duration of analgesic consumption did not show any significant differences, but a trend did emerge. Patients with rCPIP needed analgesics for an average of 5.3 days, whereas patients without CPIP took them for 2.2 days and patients with pCPIP for only 2 days (*p* = 0.062) (Fig. 2b). There was a significant difference in the length of hospital stay, although it must be borne in mind that all rCPIP patients underwent outpatient surgery. Patients with pCPIP showed a significantly longer hospital stay (2 days) than patients with rCPIP (0 days, outpatient) and patients with no CPIP (0.36 days; *p* < 0.001) (Fig. 2c).

### Potential advantages of a caudal epidural block

The subgroup analysis of an intraoperative caudal epidural block versus no caudal epidural block showed several possible positive aspects in favor of a block. Postoperative pain levels on the VAS at rest and during movement were significantly lower (*p* < 0.001) in patients with intraoperative caudal epidural block. In accordance, a significantly shorter duration of analgesia was required postoperatively in patients after caudal epidural block (2.1 days vs. 6.7 days; *p* < 0.001). Moreover, patients with a caudal epidural block showed a marginally significant shorter hospital stay (0.37 days vs. 0.86 days; *p* = 0.051). Persistent CPIP was also significantly lower in patients with caudal epidural block (*p* < 0.001). Only 2.4% of patients showed a pain duration of more than one year, whereas 28.6% of patients without a caudal epidural block had pain for more than one year postoperatively. (Table 3).

**Table 3:** Subgroup analysis of cauda epidural block patients

	No cauda epidural block = 7 (4%)	Cauda epidural block = 168 (96%)	p-value
Operating time [11] MV (SD)	35.9 (11.4)	27.2 (21.2)	0.288
Pain medication n (%)			0.451
No	3 (42.9)	70 (41.7)	
Ibuprofen	1 (14.3)	59 (35.1)	
Paracetamol	0	9 (5.4)	
Metamizole	0	2 (1.2)	
Opioids	0	3 (1.8)	
Combination	3 (42.9)	25 (14.9)	
Days in hospital MV (SD)	0.86 (0.9)	0.37 (0.6)	**0.051**
CPIP n (%)			**0.001**
No cpip	5 (71.4)	164 (97.6)	
Resolved	1 (14.3)	2 (1.2)	
Cpip	1 (14.3)	2 (1.2)	
Analgesics score MV (SD)	0.29 (0.5)	0.33 (0.6)	0.855
Duration of pain n (%)			**0.001**
< 1 week	2 (28.6)	119 (72.1)	
1 week–1 month	3 (42.9)	39 (23.6)	
1–3 month	0	3 (1.8)	
4–6 month	0	0	
7–12 month	0	0	
> 1 year	2 (28.6)	4 (2.4)	
Pain level MV (SD)			
At rest	0.71 (1.3)	0.03 (0.3)	**0.001**
On movement	1.14 (2.0)	0.01 (0.1)	**0.001**
Duration of analgesic use [days] MV (SD)	6.7 (3.6)	2.1 (2.0)	**0.001**
Incapacity of the parents [days] MV (SD)	0.86 (1.9)	2.9 (2.8)	0.062

## Discussion

Our study population showed a prevalence of 3.4% for the development of CPIP after inguinal hernia repair. This is in line with the prevalences already described in the literature (1–5%) [2, 3]. Compared to adults, who show a CPIP rate after surgical repair of inguinal hernias of approx. 10–14% [14, 17], the prevalence is significantly lower. However, in pediatric CPIP, resolution appears lower than in adults (50% vs. 70%) [9]. In summary, CPIP is less common in children, but has a higher risk of persistence.

Compared to previous studies, our follow-up period was shorter, focusing on the acute phase [10, 11]. Importantly, this allowed to distinguish between resolved and persistent CPIP.

By analyzing the retrospective data, several potential risk factors for CPIP were identified in this study: older age at operation, female gender and a history of chronic pain. Young age and female gender are reported as risk factors for the development of CPIP in adults after inguinal hernia repair [18].

In this study, the Analgesics score as a known tool to enable comparison of chronic postsurgical pain could not be evaluated as a predictor of CPIP after inguinal hernia repair in children [14]. As surgical treatment was usually performed on an outpatient basis, no information on type and dosage of analgesics after discharge could be obtained. An attempt close this gap by retrospectively interviewing the parents yielded fruitless, due to inaccuracies. A reliable statement can be made only about the duration of postoperative analgesia after discharge. In principle, patients who develop a CPIP have a longer duration of pain medication intake due to the longer-lasting postoperative pain. In our study cohort patients with rCPIP took pain medication for a significantly longer period postoperative than patients with pCPIP. The differences between rCPIP and pCPIP patients in the duration of use point towards a protective and resolving effect of adequate analgesia after discharge.

Previous studies have already shown that causa epidural blocks are an effective option for analgesia in the context of inguinal hernia repair in children, resulting in lower postoperative pain levels [19, 20]. As a result, systematic analgesics can be spared, resulting in less side effects [21]. This was confirmed by our subgroup analysis on caudal epidural block. Interestingly, the caudal epidural block might protect against CPIP development: In our study, CPIP prevalence was significantly reduced after intraoperative CEB. This finding is in line with previous findings and should be confirmed in a prospective study [22].

In summary, our study not only confirmed the lower prevalence rates of CPIP in children but detected putative factors regulating CPIP emergence. Thus, the routine use of CEB, short surgical intervention, and adequate postoperative analgesia might help to further reduce CPIP prevalence in children.

### Strength and limitations

The strength of this study is the representation of real-life conditions and thus reliable answers to the questions. The main limitations of our study are its retrospective nature and single institution design. In addition, the sample of patients with CPIP is significantly smaller due to its low prevalence. Thus, all inferential statistics of this group must be interpreted with caution. Therefore, a prospective study with structured observation would have to be carried out to clarify these indications. We plan to start this study in the next year and want to encourage other centers to take part in this study.

## Conclusion

The aim of this study was to investigate the prevalence of CPIP in children after open inguinal hernia surgery and to identify possible risk factors and protective factors for the development of CPIP. In our study population, the structured follow-up showed a CPIP rate of 3.4%. Initial risk factors such as female gender, older patient age and a history of chronic pain were identified. In addition, we found indications of possible protective factors such as an intraoperative caudal epidural block and adequate postoperative analgesia. However, further studies are required to clarify the pathogenesis and to confirm predictors and protective factors in order to improve therapeutic approaches.

## Electronic supplementary material 

Below is the link to the electronic supplementary material.

Supplementary material 1 (DOCX 120kb)
