# Supplementary material for: Chronic postoperative inguinal pain (CPIP) after pediatric inguinal hernia repair—a retrospective analysis
Source: Hernia. 2025 Jan 6;29(1):62. doi: 10.1007/s10029-024-03245-z (PMC11700912; doi:10.1007/s10029-024-03245-z)
Supplement: Supplementary file 1 — Supplementary material 1 (DOCX 120kb) [file 10029_2024_3245_MOESM1_ESM.docx]

Interview

„Long-term results after surgical treatment of a paediatric inguinal hernia”

My name is…. and I work at the Department of Surgery at the University Hospital of Wuerzburg.

Your child was operated on …………. due to a inguinal hernia.

You kindly returned you declaration of consent to participate in our study. Thank you very much for you willingness to support our study. In this short interview we would like to find out about your child’s current state of health and how things are going after the operation.

1. How are you and your child (individual answer)
2. Does your child still have pain after the operation
   1. Yes
   2. No
3. If no, How ling did your child have pain after the operation
   1. Up to one week
   2. One week – one month
   3. One – three months
   4. Four – six months
   5. Seven – twelve months
   6. Longer than one year (till now)
4. If your child still complains about pain
   1. How severe was the pain at maximum during the last week?


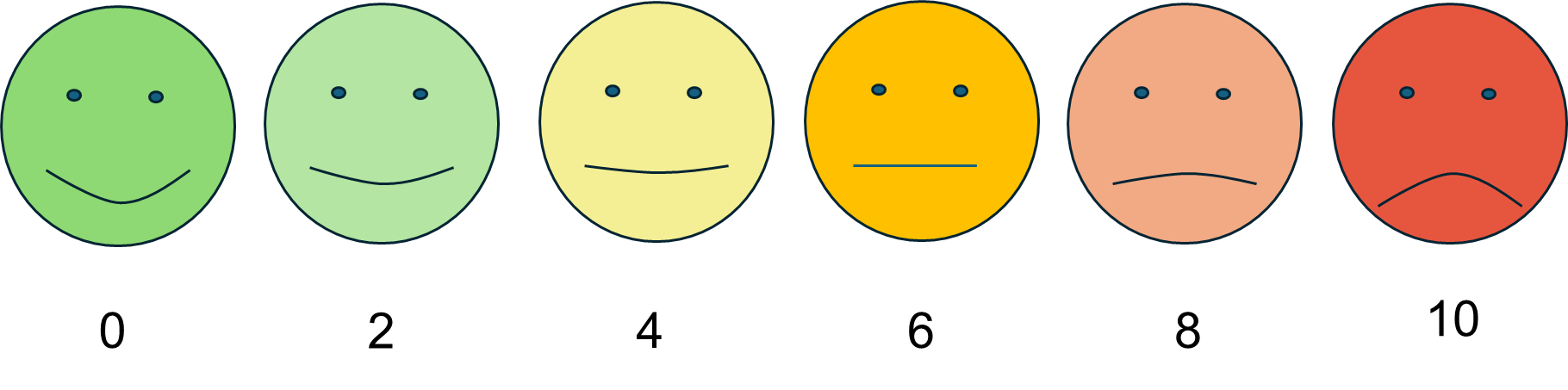


- 1. How severe was the pain at average during the last week?


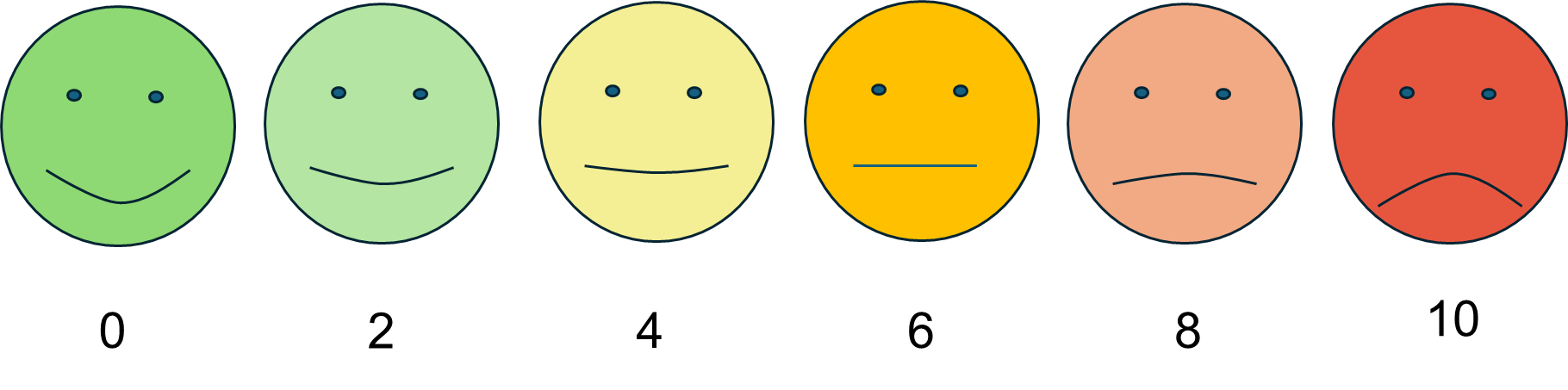


- 1. How was the pain playing and under heavy load?


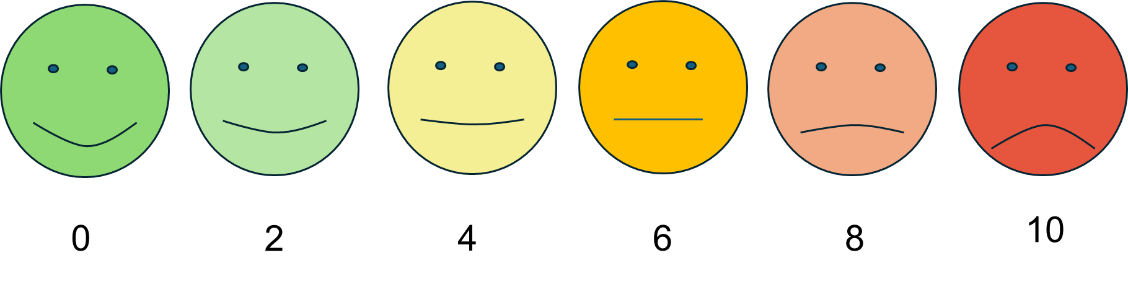


1. How does your child describe the pain?

a. My pain is deep inside and presses (dull)

b. My pain stings like a needle (sharp/stabbing)

c. My pain comes all of a sudden (shooting in)

d. My pain throbs like my heart (pulsating)

e. My pain burns like fire or stinging nettles

f. My pain causes everything to contract (cramp-like)

1. What does you child do when it is in pain?
2. What do you do, when your child is in pain?
3. How long did your child take pain killers after the operation?
4. How long were you as parents affected by the operation, or how many days were you absent from work because of the operation?
5. How long was your child absent from the kindergarten / school

We would like to thank you very much for your time and your participation in our survey.
